# Determinants of Behavioral Intention to Use Digital Healthcare Services: A Cross‐Sectional PLS‐SEM Study on the Shukhee App in Bangladesh

**DOI:** 10.1002/hsr2.73224

**Published:** 2026-09-13

**Authors:** Shanjida Chowdhury, Farhana Ferdousi, Tahsin Farzana Jisun

**Affiliations:** ^1^ School of Business Studies Southeast University Dhaka Bangladesh; ^2^ Faculty of Business Administration (FBA) American International University‐Bangladesh Dhaka Bangladesh

**Keywords:** e‐health, Grameen digital health services, Shukhee, social business, TAM, UTAUT

## Abstract

**Background and Aims:**

The rapid proliferation of digital technologies in the healthcare system has made digital health services indispensable for enhancing access, efficiency, and patient engagement. Digital healthcare, such as the Shukhee app, has become prominent; however, the factors influencing users' behavioral intention to adopt it have not been studied extensively. This paper aims to examine the important variables that determine the behavioral intention to use digital health services with specific focus on the mediating effect of e‐health literacy.

**Methods:**

This paper adopts the Technology Acceptance Model (TAM) and the Unified Theory of Acceptance and Use of Technology (UTAUT) to determine the factors that affect the behavioral intention to use digital health services, with a specific focus on the mediating effect of e‐health literacy. A total of 417 digital health service user or were familiar with the services within the past year were chosen in this study. Data were analyzed using partial least squares‐based structural equation modeling (PLS–SEM) with SmartPLS 4.1.

**Results:**

The findings show that perceived trust, social influence, performance expectancy, facilitating conditions, effort expectancy, and e‐health literacy significantly predicted behavioral intention. E‐health literacy also mediates the impact of critical predictors like trust, social influence, and performance expectancy, while not showing significant mediation for perceived ease of use, perceived usefulness, effort expectancy, or facilitating conditions.

**Conclusion:**

The intention to use digital healthcare services is influenced by technology‐related perceptions, as well as trust, social context, and digital health competencies of users. The results highlight that enhancing trust, empowering social support, and promoting e‐health literacy can be particularly significant for Bangladesh and other settings in improving the uptake of digital healthcare platforms.

**Abbreviations**
AVE: average variance extracted
CI: confidence interval
*f* ^2^
: effect size for a predictor in PLS‐SEM
HTMT: heterotrait–monotrait ratio
PLS‐SEM: partial least squares structural equation modeling
*Q* ^2^
: predictive relevance
*R* ^2^
: coefficient of determination
SD: standard deviation
SE: standard error
TAM: technology acceptance model
UTAUT: unified theory of acceptance and use of technology
VIF: variance inflation factor
*β* (beta): standardized path coefficient indicating the strength and direction of a relationship

## 1 Introduction

The global digitalization of health care systems has radically transformed how health information is accessed, interpreted, and managed. Technologies like telemedicine, m‐health applications, patient portals, and digital health information platforms are becoming crucial for enhancing efficiency, patient‐centered care, and accessibility for underserved populations [1, 2, 3]. As digital health infrastructure has accelerated, healthcare delivery is becoming more dependent on remote consultations and technology‐mediated care and online access to health information and records [4, 5].

Despite rapid digitalization, adoption of healthcare services remains uneven. Existing disparity in uptake of e‐health services across and within populations continues to be influenced by digital literacy gaps and usability issues [6, 7], privacy and security concerns [8, 9], cultural, demographic, and contextual factors [9, 10, 11], and perceived risk and technophobia [7, 12]. This inconsistency highlights the essence of crucial drivers that shape behavioral intentions to adopt digital health care services, particularly as digital tools become integrated into routine healthcare provision.

Among various determinants, e‐health literacy is a crucial determinant of influencing the adoption of digital health care services. E‐health literacy refers to an individual's ability to seek, evaluate, comprehend, and use electronic health information [13, 14]. People with higher e‐health literacy demonstrate higher confidence in navigating digital environments, enhanced understanding of health‐related information, and a higher likelihood of adopting e‐health services [15, 16]. Conversely, those with lower e‐health literacy struggle with issues such as encountering misinformation, insufficient ability to differentiate between trustworthy sources, and a lack of motivation to seek help using electronic mediums [17, 18, 19]. The implication of e‐health literacy is also dependent on the digital competencies of healthcare providers [20, 21, 22]. E‐health literacy is therefore not only a technical skill but also a capability that may shape whether digital healthcare services are considered as useful, manageable, and trustworthy. Recent studies suggest that e‐health literacy can influence the determinants of psychological importance, perceived ease of use, and perceived usefulness, which in turn further impact behavioral intention [15]. However, the results remain inconsistent across populations and technological settings [23, 24, 25, 26]. These findings highlight the importance of further in‐depth analysis of the role of literacy within broader models of technology adoption, especially in low‐ and middle‐income countries settings.

In order to better understand the factors influencing technology adoption, previous research has commonly applied established theoretical frameworks such as the Technology Acceptance Model (TAM) and the Unified Theory of Acceptance and Use of Technology (UTAUT). The idea of TAM is to consider perceived usefulness and perceived ease of use as the major factors of technology adoption, but UTAUT introduces the concept of performance expectancy, effort expectancy, social influence, and facilitating conditions. These models have been widely validated in healthcare settings, but the strength of their explanation may also differ based on the context where users face substantial informational, technological, or social barriers.

Bangladesh provides an important and timely context to examine the determinants of behavioral intention to utilize the digital healthcare services. As a lower‐middle‐income country LMIC undergoing digital transformation, Bangladesh has made significant progress in expanding mobile connectivity, internet access, and digital health initiatives. But the proportion of the population accessing digital health systems remains low, with high‐income and educated people disproportionately represented on digital platforms. In Bangladesh, only 7.2% of device owners used them for health, with lower use among women, the less educated, and the poor. Barriers include a lack of awareness, skills, low digital health literacy, and resource constraints that continue to restrict equitable access to digital healthcare services [27]. Within this context, a digital healthcare platform such as the *Shukhee*, which is launched by Grameen HealthTech Limited, has emerged to address gaps in healthcare accessibility. Regardless of potential, little empirical research has been conducted on the factors that shape the behavioral intent of users to adopt such platforms in Bangladesh. This paper addresses that gap by examining behavioral intentions toward digital healthcare services, specifically the Shukhee (app) platform, using a combination of TAM and UTAUT, with particular emphasis on the mediating role of e‐health literacy. The novelty of this study lies in its specific focus on the digital health care services, that is, Shukhee platform. Researchers also consider e‐health literacy both as a direct predictor and a mediator, while also integrating TAM and UTAUT models in a context where digital health adoption remains underexplored, particularly in LMICs.

### 1.1 Literature Review

Digital healthcare services are becoming increasingly popular worldwide, aiming to expand access to healthcare and enhance patient engagement. The adoption of these services is still inconsistent despite its advantages. Digital literacy [28], trust [29], privacy issues [30], perceived risk [29, 31], and social factors [28, 32] are just some factors that influence the decisions of individuals to adopt digital health solutions. These issues are especially notable in low‐ and middle‐income countries (LMICs), as health disparities may be increased through digital illiteracy and barriers to technology.

For understanding the process of digital technology use in healthcare, two notable models are the Technology Acceptance Model (TAM) and the Unified Theory of Acceptance and Use of Technology (UTAUT). They have been popular models for investigating the determinants of technology adoption, even in the digital health setting. Within TAM, perceived ease of use (PEOU) refers to the extent to which users believe that a system is effortless to use and operate, whereas perceived usefulness (PU) reflects that the use of the system will enhance health management or daily life.

Based on the UTAUT approach, performance expectancy (PE) is an important element in promoting the utilization of digital health services. Users will embrace the use of digital health platforms more when they feel that they are helpful in managing their health or improving their results. This is in line with the overall studies of adoption of technology, whereby the decision to adopt new technology is usually affected by the perceived usefulness of the technology [33, 34]. The effort expectancy (EE) factor is also critical, particularly to those users who are not familiar with digital technologies. Users will be able to use a user‐friendly platform that does not require much effort to learn. Therefore, accessibility and ease of use should be considered as well to make digital health platforms accessible to elderly citizens or individuals with low levels of technical proficiency [34]. The following Table 1 summarizes key studies that have applied both TAM and UTAUT to investigate determinants of behavioral intention to use digital healthcare services.

**TABLE 1:** Summary of studies using both TAM and UTAUT to analyze behavioral intention in digital healthcare.

Study	Key determinants identified (TAM and UTAUT)	Methodology (PLS‐SEM/other)
EMR adoption in UAE [35]	TAM: Perceived usefulness, ease of use, trust, innovativeness, self‐efficacy; UTAUT: Performance expectancy, effort expectancy, social influence, facilitating conditions	PLS‐SEM, ANN
Mobile EMR in tertiary hospital [36]	Performance expectancy, attitude, facilitating conditions, social influence (TAM and UTAUT constructs combined)	SEM, log file analysis
Older adults' use of medical apps (COVID‐19, China) [37]	Attitude, perceived usefulness, facilitating conditions, perceived ease of use, subjective norm (TAM and UTAUT)	SEM
Systematic review of remote healthcare tech acceptance [34]	Studies use integrated TAM and UTAUT; both models are valid for predicting acceptance	Systematic review
Review of psychosocial barriers in healthcare tech [38]	Performance expectancy, effort expectancy, social influence, trust, perceived risk, attitude (TAM and UTAUT)	Review, SPSS/AMOS
Technology acceptance in health informatics [39]	Both models predict adoption; context‐specific, socio‐organizational factors important	Case studies
mHealth app usage (Indonesian users) [40]	Effort expectancy, performance expectancy, social influence (UTAUT) → individual belief → intention to use (TAM/UTAUT)	SEM (Smart‐PLS)
Digital health for NCDs in older adults [41]	TAM and UTAUT most frequent frameworks; perceived usefulness, ease of use, social influence, e‐health literacy	Systematic review

Author's Compilation

As shown in Table 1, various previous studies have applied TAM and/or UTAUT to determine digital health care adoption. Very little research has incorporated e‐health literacy as a predictor [41, 42]. None of these studies, however, were carried out in a low‐ or middle‐income country (LMIC) such as Bangladesh and not on a social business health app like Shukhee. Besides, none of them combined all the TAM and UTAUT constructs with e‐health literacy as a direct predictor, as well as a mediator. Our research paper bridges this knowledge gap by presenting empirical data of 417 Shukhee users in Dhaka, Bangladesh.

Previous research tends to state that users perceive digital health platforms as easy and helpful, and therefore, they are more likely to have an intention to use them [29, 43, 44]. These relationships remain central in healthcare technology adoption, even though their strength may vary across contexts and user groups. Based on this evidence, the study anticipates that PEOU and PU will positively influence behavioral intention to use digital healthcare services. Therefore, the following hypotheses are proposed:

H1: Perceived Ease of Use → Behavioral Intention to Use;

H2: Perceived Usefulness → Behavioral Intention to Use.

Studies on telemedicine, mHealth, and patient‐facing digital services have demonstrated that the willingness to apply these technologies rises when the users anticipate meaningful benefits and manageable effort [38, 40, 45]. In the context of healthcare, these expectations are particularly applicable as users consider the practical usefulness of the technology, as well as the learning or usage burden [46]. Thus, it is reasonable to predict that PE and EE will positively affect behavioral intention. So, the study proposes the following hypotheses:

H3: Performance Expectancy → Behavioral Intention to Use;

H4: Effort Expectancy → Behavioral Intention to Use.

Social influence (SI) is a significant factor that affects individuals' intentions to adopt digital health services. Social influence from family, friends, media, and health professionals significantly increases intention to use telemedicine, mHealth apps, and other e‑health tools [34, 47]. Similarly, facilitating conditions (FC) refer to the extent to which individuals perceive that there is organizational, technical, and financial assistance to aid them in utilizing the digital healthcare platform. Previous studies speculate that support, resources, and alignment with the circumstances of users can bolster the intention to adopt digital health tools [2, 44]. Therefore, this study assumes positive effects of both SI and FC on behavioral intention. The following hypotheses are proposed:

H5: Social Influence → Behavioral Intention to Use;

H6: Facilitating Conditions → Behavioral Intention to Use.

Besides the original TAM and UTAUT constructs, perceived trust (PT) has emerged as a key determinant in digital healthcare use. Since online health systems deal with very sensitive personal data, their users should be convinced that the system is safe, reliable, and able to facilitate proper healthcare practices. Previous research has always demonstrated that there is a close relationship between trust and the intention to use e‐health, telemedicine, and mobile health applications [48, 49]. Users will be more likely to adopt a platform when they find it to be trustworthy and therefore, come over with uncertainty. Based on this, the paper hypothesizes:

H7: Perceived Trust → Behavioral Intention to Use.

Another concept that has been gaining more and more attention is e‐health literacy (EL) which can be described as the skill to locate, comprehend, analyze, and apply health data available online. The significance of EL is in the fact that the successful utilization of digital healthcare services not only depends on access to a platform but also on the ability of the users to process information and take actions. Previous studies indicate that individuals with higher e‐health literacy are associated with greater confidence in using digital platforms and intention to use digital health services [11, 17, 50]. This research will, thus, anticipate a positive and direct impact of EL on behavioral intention. In this way,

H8: E‐Health Literacy → Behavioral Intention to Use is proposed.

In addition to its direct impact, EL can also impact as a mediating factor between technology‐related perceptions and behavioral intention. The rationale is that positive attitudes toward a digital health platform might not be developed into intention completely unless users also have the capacity and confidence to interact with digital health information [13, 14, 16]. Illustratively, users who feel that a platform is useful, easy to use, trustworthy, or socially approved may reinforce their e‐health literacy or their productive interaction with digital health information, consequently increasing behavioral intention. Available research indicates that mediation of this type is feasible, but there is empirical evidence that is inconsistent in the contexts [13, 14, 16]. Based on this, the current study explores the issue of EL, whether it mediates the connection between the primary antecedents and behavioral intention. Based on this, the following mediation hypotheses are postulated:

H9a: Perceived Ease of Use → E‐Health Literacy → Behavioral Intention to Use;

H9b: Perceived Usefulness → E‐Health Literacy → Behavioral Intention to Use;

H9c: Performance Expectancy → E‐Health Literacy → Behavioral Intention to Use;

H9d: Effort Expectancy → E‐Health Literacy → Behavioral Intention to Use;

H9e: Social Influence → E‐Health Literacy → Behavioral Intention to Use;

H9f: Facilitating Conditions → E‐Health Literacy → Behavioral Intention to Use; and

H9g: Perceived Trust → E‐Health Literacy → Behavioral Intention to Use.

**FIGURE 1:**
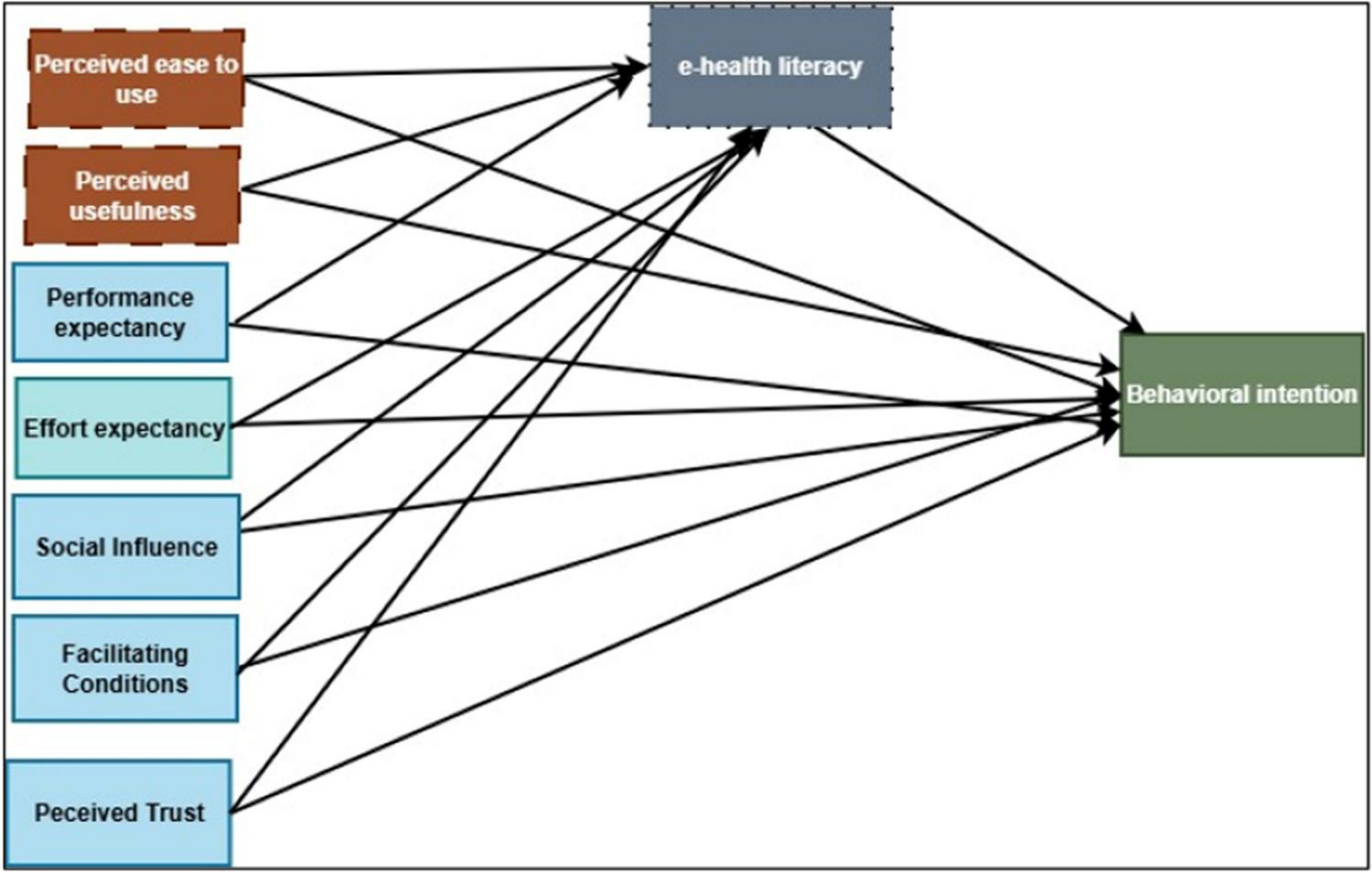
Integrated TAM and UTAUT conceptual model of behavioral intention to use digital health care services.

Drawing on the literature reviewed above, the paper's research model is presented in Figure 1, which integrates the TAM and UTAUT models extended with perceived trust and e‐health literacy to capture the determinants of digital health adoption. Specifically, perceived ease of use (PEOU) and perceived usefulness (PU) are drawn from TAM, which represent core perceptions of usability and benefits. UTAUT constructs are performance expectancy (PE), effort expectancy (EE), social influence (SI), and facilitating conditions (FC), which reflect motivational, social, and contextual influences. Moreover, perceived trust is added as an external antecedent, and e‐health literacy serves both as a direct predictor and as a mediator. Our outcome variable is behavioral intention to use.

## 2 Methodology

### 2.1 Participants

This study used purposive sampling to select respondents who were experienced or knowledgeable in digital healthcare services. Inclusion criteria include respondents aged 18 or older residing in Dhaka, Bangladesh, who had used or were familiar with the Shukhee app, a popular e‐health application that provides telemedicine, medical information, and healthcare access. Eligible participants had to provide consent to complete the questionnaire and answer five pre‐test questions about basic application functions covering basic information, utilities, trustworthiness, and personal experience, to confirm familiarity with the application. In total, 450 responses were collected online via Google Form. After removing 33 responses (duplicates, responses with missing data, and those who answered the basic pre‐test questions incorrectly), 417 responses from Dhaka were retained, representing a response rate of 92.7% (417/450). The survey questionnaire was distributed through multiple online channels (social media and email), so the total number of individuals exposed to the invitations could not be determined. Non‐response bias is solved using a pre‐ and post‐stage *t*‐test. The test showed no statistically significant difference between the two groups (*t* = 1.286, *p* > 0.05), representing no non‐response bias. Dhaka was chosen for its status as the nation's center and for facilitating access to diverse participant backgrounds. Data were collected from July 1, 2025, to September 15, 2025.

### 2.2 Ethical Considerations

The ethical review committee of Southeast University, Bangladesh, approved this study (SEU/IRT/Ethical Approval/2025/06/29‐08). The purpose of the study and the voluntary nature of participation were explained to participants before they participated in the survey. Also, informed consent was obtained from all participants in accordance with the Declaration of Helsinki before they responded to the questionnaire. Data were collected anonymously, stored securely and used solely for academic research.

### 2.3 Questionnaire Development

The questionnaire was designed by adapting and refining constructs from the available literature, thereby establishing its contextual relevance to digital healthcare services. The instrument consisted of 10 key sections, each corresponding to a specific construct identified in the research. In the first part, demographic information was gathered, such as the age of participants, their gender, and their levels of education. The second part, which focused on the perceived ease of use, challenged the perception of the participants toward the technical usability of the Shukhee platform and the learning effort they perceived using the platform. The third part, concerned with the perceived usefulness, tested the perceived benefits, efficiency, and positive effects that were expected with the use of Shukhee platform. Section 4 was about social influence, which investigated the level of influence peers, family members, and colleagues have on the usage of the Shukhee platform by the participants. The fifth section on Facilitating conditions assessed perceptions of technical support and financial resources. The sixth section focused on Performance expectancy, which refers to the extent to which users believe that using the Shukhee will enhance their job performance, productivity, or work‐related outcomes. The following section is Effort expectancy, which assesses clarity, learning effort, and required skills. The eighth section measures perceived trust, which reflects the belief that the system performs correctly and can be relied upon. The ninth section demonstrates the user's ability to find, understand, evaluate, and use online health information effectively, while the final section focuses on behavioral intention or willingness to continue using the Shukhee in the future. Details of items presented in Table 2.

**TABLE 2:** Description of the items in the construct.

Construct	Items
Perceived usefulness [39, 51] (PU)	PU1 Shukhee platform will help me manage my health.
	PU2 I believe that using Shukhee platform will make my daily life safe.
	PU3 Using Shukhee platform will improve my quality of life.
Perceived ease to use [39, 51] (PEOU)	PEOU1 I think using Shukhee platform would be simple.
	PEOU2 Learning to use Shukhee platform will be easy.
	PEOU3 Shukhee platform will be convenient to use.
Performance expectancy [42, 52] (PE)	PE1 I believe that Shukhee platform will be useful in my work.
	PE2 Using Shukhee platform will enable me to accomplish work tasks more quickly.
	PE3 Using Shukhee platform will increase my productivity.
	PE4 Using Shukhee platform will improve my job performance.
Effort expectancy [42, 52] (EE)	EE1 My interaction with Shukhee platform will be clear and understandable.
	EE2 It is easy for me to become skillful at using Shukhee.
	EE3 I believe that Shukhee platform will be easy to use.
	EE4 Using Shukhee platform will be easy for me.
Social influence [42, 52] (SI)	SI1 People who influence my behavior think I should use Shukhee.
	SI2 People who are important to me think I should use Shukhee.
	SI3 People who are important to me recommend me to I should use Shukhee.
Facilitating conditions [42, 52] (FC)	FC1 I will know how to use the Shukhee.
	FC2 If I encounter difficulties using your Shukhee, I think someone will be able to help.
	FC3 I have sufficient financial resources to use Shukheec.
Behavioral intention [42, 52] (BI)	IU1 I intend to continue using Shukhee platform to manage my health in the future.
	IU2 My willingness to use Shukhee platform is high.
	IU3 I plan to continue to use Shukhee platform in the future.
e‐Health literacy [14] [11, 17, 50] (EL)	EL1 I know what health resources are available on the Internet.
	EL2 I know where to helpful health resources on the Internet.
	EL3 I know how to helpful health resources on the Internet.
	EL4 I know how to use the Internet to obtain answers to my questions about health.
	EL5 I know how to use health information on the Internet to help me.
	EL6 I can rate the health resources on the Internet.
	EL7 I can distinguish between high‐ and low‐quality health resources on the Internet.
	EL8 I can determine the correctness of health information on the Internet.
	EL9 I feel confident in using information from the Internet to make health decisions.
Perceived trust [48] (TR)	TR1 The Shukhee platform works reliably.
	TR2 I am confident about the capabilities of Shukhee platform.
	TR3 The Shukhee platform is capable of interpreting situations correctly.
	TR4 I trust the Shukhee platform.
	TR5 I can rely on the Shukhee platform.

### 2.4 Data Analysis

The reliability and validity of the questionnaire were checked by the use of Cronbach's alpha. To justify non‐response bias, this study also divided the sample size into two stages of data collection (pre‐stage: *n*_1_ = 208; post‐stage, *n*_2_ = 209) and executed a *t*‐test, resulting in a statistically insignificant *p*‐value (*t*‐statistic: 1.286; *p*‐value: > 0.05). Hence, plausible chances of response bias are not observed. To check whether data follows a normal distribution, this study check if the values for skewness and kurtosis fall within ±2 and ±7, respectively [53, 54]. For checking multicollinearity, the most applicable single‐factor test according to the procedure of Harman (1976) was applied to address common method variance (CMV) among the constructs [55] and it observed single factor variation is 47.4% which is less than cut‐point 50.0%. To ensure efficiency and sufficiency of the model, collinearity diagnostics were strictly undertaken [56]. The variance inflation factor (VIF) result suggests that there is no issue of multicollinearity [57]. The analysis process, after reviewing the data, was conducted using Structural Equation Modeling (SEM), which approximates causal relationships by combining statistical data and quantitative causal hypotheses. Statistical reporting followed the principles of transparent reporting recommended by Assel et al. [58], including demonstration of exact *p*‐values, 95% confidence intervals for all path coefficients. To assess the discriminant validity, this study used the Fornell–Larcker criterion and the Heterotrait–Monotrait Ratio (HTMT) [54, 59, 60]. We considered *f* ^2^ effect sizes, *Q* ^2^ predictive relevance, and *R* ^2^ values to measure the structural model. The *f* ^2^ effect sizes were used to determine the strength of each predictor's impact where small (*f* ^2^ = 0.02), medium (*f* ^2^ = 0.15), and large (*f* ^2^ = 0.35) [61]. *Q* ^2^ predictive relevance was measured via blindfolding to assess the model's predictive accuracy, with values greater than 0, which suggest significant predictive relevance [54]. *R* ^2^ values represented the proportion of variance explained by the model and were classified as small (*R* ^2^ < 0.25), moderate (0.25 ≤ *R* ^2^ < 0.50), or substantial (*R* ^2^ ≥ 0.50) [62]. All the tests were two‐tailed with *α* = 0.05. For reporting follows SAMPL guidelines. SmartPLS 4.1 was used to estimate the research model [63] as it can handle missing data and small sample sizes. This study used only the reflective model. The former evaluation discipline involved first assessing the reliability and validity of the measurement model, followed by evaluating the structural model.

## 3 Results

Table 3 presents the demographic characteristics of the sample. Among the 417 respondents, 247 (59.0%) reported prior experience with digital health care services, specifically the Sukhee app. The sample comprised 252 male and 165 female participants. A total of 129 participants were aged 20–29 years, representing 39.6% of the sample. The majority of respondents held a bachelor's degree (48.0%). Appendix Table A1 provides descriptive statistics for nine constructs (42 items). On the 5‐point response scale, mean scores ranged from 3.11 (BI02) to 3.54 (PE01). BI02 and PE01 received the highest mean ratings from users who reported positive experiences with the services. Standard deviations ranged from 1.21 to 1.40, indicating adequate variability in responses. Skewness values ranged from −0.654 to −0.133, and kurtosis values ranged from −1.102 to −0.576, both within the accepted ranges of ±2 for skewness and ±7 for kurtosis.

**TABLE 3:** Demographic statistics.

Variables	Categories	*N* (%) (*n* = 417)
Digital healthcare experience (Sukhee)	Use	247 (59.2)
	Heard but not used	170 (40.8)
Gender	Male	252 (60.4)
	Female	165 (39.6)
Age	20–29	129 (30.9)
	30–39	120 (28.8)
	40–49	98 (23.5)
	50+	70 (16.8)
Education	Higher secondary or less	72 (17.2)
	Bachelor's degree	200 (48.0)
	Master's or higher	145 (34.8)

### 3.1 Measurement Model Assessment

There was a strong reliability and validity in the measurement model. All the constructs had significant internal consistency with the Cronbach's alpha values between 0.773 and 0.899, which are well above the traditional level of 0.70. Measurements of composite reliabilities ranged between 0.855 and 0.918, thus indicating good internal consistency reliability. Additional evidence of the adequate convergent validity was the average variance extracted (AVE) values, which ranged between 0.553 and 0.762, hence validating the adequate convergent validity. Table 4 presents the assessment result.

**TABLE 4:** Convergent validity analysis.

	Cronbach's alpha	Composite reliability (rho_a)	Composite reliability (rho_c)	Average variance extracted (AVE)
Behavioral intention to use (BI)	0.845	0.846	0.896	0.683
E‐health literacy (EL)	0.899	0.900	0.918	0.554
Effort expectancy (EE)	0.773	0.774	0.855	0.595
Facilitating conditions (FC)	0.781	0.783	0.859	0.603
Perceived ease of use (PEOU)	0.775	0.777	0.855	0.597
Perceived trust (TR)	0.838	0.839	0.881	0.553
Perceived usefulness (PU)	0.777	0.779	0.857	0.599
Performance expectancy (PE)	0.844	0.845	0.906	0.762
Social influence (SI)	0.878	0.878	0.916	0.731

#### 3.1.1 Convergent Validity

The items' factor loadings (outer loadings) ranged from 0.699 to 0.881, and the variance inflation factors (VIFs) ranged from 1.432 to 2.225; hence, no multicollinearity issues (results shown in the Appendix Table A2). These findings support convergent validity, as all items showed strong agreement with their intended constructs.

#### 3.1.2 Discriminant Validity

Using the Fornell–Larcker (Table 5) criterion, the square roots of the values of the AVE were higher than the inter‐construct correlations of all constructs. In this study, behavioral intention to use (AVE = 0.827) exceeded the correlations with other constructs, confirming satisfactory discriminant validity across all latent variables. In contrast, cross‐loadings represent the relationships between each indicator variable and all latent constructs within the measurement model (as shown in the Appendix Table A2). Evaluating cross‐loadings is essential for establishing discriminant validity at the individual indicator level, together with the Fornell–Larcker criterion.

**TABLE 5:** Fornell–Larcker criterion.

	BI	EL	EE	FC	PEOU	TR	PU	PE	SI
BI	0.827								
EL	0.761	0.744							
EE	0.770	0.695	0.772						
FC	0.800	0.722	0.675	0.777					
PEOU	0.777	0.677	0.654	0.766	0.773				
TR	0.772	0.644	0.612	0.666	0.669	0.744			
PU	0.786	0.679	0.654	0.756	0.652	0.672	0.774		
PE	0.733	0.633	0.676	0.680	0.678	0.633	0.692	0.773	
SI	0.746	0.650	0.695	0.684	0.618	0.678	0.688	0.762	0.755

### 3.2 Structural Model Assessment

#### 3.2.1 Direct Effects

The results (Table 6) reveal that perceived trust, social influence, facilitating conditions, performance expectancy, effort expectancy, and e‐health literacy significantly increase users' intention to engage with e‐health services.

**TABLE 6:** Path analysis and hypothesis testing.

Direct effects	Original sample	SD	*t*‐statistics	*p*‐values	*f* ^2^	2.5% C.I.	97.5% C.I.
H1: Perceived Ease of Use ‐> Behavioral Intention to Use	0.060	0.042	1.437	0.151	0.005	−0.022	0.141
H2: Perceived Usefulness ‐> Behavioral Intention to Use	0.076	0.045	1.685	0.092	0.008	−0.012	0.164
H3: Performance Expectancy ‐> Behavioral Intention to Use	0.121	0.054	2.249	0.025	0.015	0.016	0.227
H4: Effort Expectancy ‐> Behavioral Intention to Use	0.096	0.039	2.472	0.013	0.014	0.019	0.172
H5: Social Influence ‐> Behavioral Intention to Use	0.191	0.059	3.265	0.001	0.030	0.082	0.313
H6: Facilitating Conditions ‐> Behavioral Intention to Use	0.119	0.043	2.74	0.006	0.019	0.032	0.203
H7: Perceived Trust ‐> Behavioral Intention to Use	0.227	0.040	5.673	0.000	0.109	0.146	0.303
H8: E‐Health Literacy ‐> Behavioral Intention to Use	0.124	0.044	2.836	0.005	0.017	0.037	0.209

The table shows the results of the hypothesis testing to verify the direct effects on behavioral intention to use based on 5000 bootstrapped subsamples using a two‐tailed test with 2.5% and 97.5% confidence intervals. Findings indicate that all the relationships are significant except for the first two. Specifically, for H1, the relationship between perceived ease of use (*β* = 0.060, *p* = 0.151) and behavioral intention to use is not significant. In H2, the significance is at the quasi‐level as the *p*‐value is less than 0.10; the effect of perceived usefulness (*β* = 0.076, *p* = 0.092) on behavioral intention to use is slightly positive. Specifically, in H3 and H4, performance expectancy (*β* = 0.121, *p* = 0.025) and effort expectancy (*β* = 0.096, *p* = 0.013) both have statistically significant effect on behavioral intention to use on 5% level of significance. Additionally, the remaining four hypotheses (H4–H8) demonstrate that social influence (*β* = 0.191, *p* = 0.001), facilitating conditions (*β* = 0.119, *p* = 0.006), e‐health literacy (*β* = 0.124, *p* = 0.005), and especially perceived trust (*β* = 0.227, *p* < 0.001) all have significant positive effects on behavioral intention to use at 1% level of significance. Moreover, the confidence intervals for these significant effects do not include 0, which further confirms their reliability. The results for the effect size measures indicate that most predictors have a small effect on behavioral intention to use (range from 0.005 to 0.030), while perceived trust has a medium‐to‐large effect on behavioral intention to use (*f* ^2^ = 0.109), accounting for 9.8% of unique variance which is substantially larger than social influence (*f* ^2^ = 0.030) and performance expectancy (*f* ^2^ = 0.015). The structural model indicates substantial explanatory power, where behavioral intentions had *R* ^2^ value of 0.815 (adjusted *R* ^2^ of 0.811) and e‐health literacy had an *R* ^2^ value of 0.799 (adjusted *R* ^2^ of 0.796). To address predictive relevance, *Q* ^2^ predict values of 0.790 for e‐health literacy and 0.804 for behavioral intentions are considerably higher than 0, as well as more than 0.50, and are considered large predictive relevance. Furthermore, the corresponding Root Mean Square Error (RMSE) for e‐health literacy (0.460) and behavioral intentions (0.444) and Mean Absolute Error (MAE) for e‐health literacy (0.360) and Behavioral Intentions (0.338) reveal minimal prediction error, thereby validating the model's predictive accuracy. All these results confirm that the model satisfies the requirements for PLS‐SEM.

**FIGURE 2:**
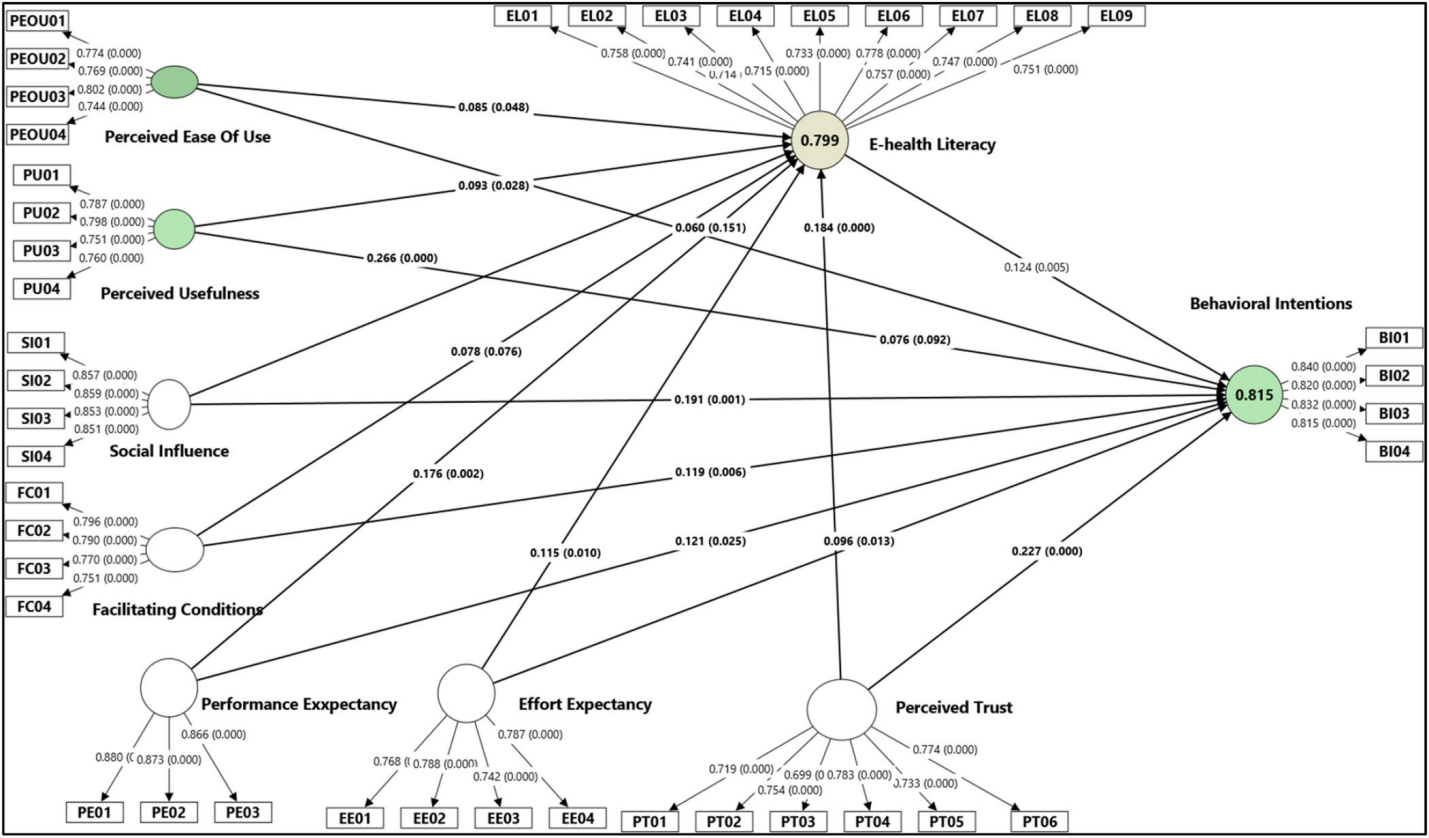
The structural model.

Given the non‐significant mediation, seven hypotheses are examined; only three are statistically significant, indicating partial mediation (shown in Table 7). Although some effects are very close to significance (e.g., Perceived Usefulness ‐> E‐Health Literacy ‐> Behavioral Intention to Use, *p*‐value: 0.085) suggesting potential mediation that may become significant in a larger sample. In other words, the significant indirect effects for performance expectancy, social influence, and perceived trust via e‐health literacy indicate partial mediation. The traditional model of mediation, as proposed by Baron and Kenny, suggested that full mediation is present when an independent variable predicts an outcome variable indirectly through the proposed mediator, and a direct relationship between the predictor and outcome variable is non‐significant. On the other hand, partial mediation occurs when there is both a significant indirect effect and a direct effect between the predictor and outcome variable [64, 65]. In this study, three predictors that had significant indirect effects (H9c, H9e, and H9g) also had significant direct effects on behavioral intention to use (H3, H5, and H7). This supports that e‐health literacy acts as an important mediator, though the magnitude of the effect is modest but it does not fully account for these relationships [66, 67]. Overall, the findings indicate that trust, performance expectation, and social influence have a significant influence on the behavioral intention in the form of increasing the e‐health literacy of users in the model first, and this proves that literacy plays a statistically significant though modest mediating role in the model. The structural model is shown in Figure 2.

**TABLE 7:** Path coefficient – mediation effect.

Indirect effect	Original sample	SD	*t*‐statistics	*p*‐values	Result
H9a: Perceived Ease of Use ‐> E‐Health Literacy ‐> Behavioral Intention to Use	0.010	0.007	1.527	0.127	No mediation
H9b: Perceived Usefulness ‐> E‐Health Literacy ‐> Behavioral Intention to Use	0.012	0.007	1.724	0.085	No mediation
H9c: Performance Expectancy ‐> E‐Health Literacy ‐> Behavioral Intention to Use	0.022	0.010	2.140	0.032	Partial mediation
H9d: Effort Expectancy ‐> E‐Health Literacy ‐> Behavioral Intention to Use	0.014	0.008	1.772	0.076	No mediation
H9e: Social Influence ‐> E‐Health Literacy ‐> Behavioral Intention to Use	0.033	0.014	2.319	0.020	Partial mediation
H9f: Facilitating Conditions ‐> E‐Health Literacy ‐> Behavioral Intention to Use	0.010	0.006	1.494	0.135	No mediation
H9g: Perceived Trust ‐> E‐Health Literacy ‐> Behavioral Intention to Use	0.023	0.010	2.247	0.025	Partial mediation

## 4 Discussion

This study employed integrated TAM and UTAUT models to examine the determinants of behavioral intention to use digital health services. Eight direct hypotheses and seven indirect hypotheses with e‐health literacy as a mediator were tested. Of the 15 hypotheses, seven direct and three indirect hypotheses demonstrated statistical significance aligned with the proposed model. The results indicate that both TAM and UTAUT have a significant role in explaining behavioral intention to use e‐health services. Among TAM constructs, only perceived usefulness shows marginal support. However, under UTAUT, perceived trust, social influence, performance expectancy, facilitating conditions, and effort expectancy play a significant role. These findings support previous studies that highlight the significance of user confidence, social reinforcement, and perceived benefits for the intention to use digital health technologies [68, 69, 70, 71]. In addition, e‐health literacy as a vital mediator of its features indicates increases in intention to use e‐health and indirectly boosts perceived usefulness and ease of use [42, 69, 72].

Perceived trust emerges as the strongest added predictor outside the core TAM and UTAUT constructs of behavioral intention to adopt e‐health services, especially when sensitive personal health information is shared or when health decisions are made digitally. This aligns with the available literature, which has found that trust is a prerequisite for adopting e‐health, especially when users are called upon to provide personal health data or to make decisions based on digital platforms [73, 74]. One of the greatest influences on e‐health literacy was trust, indicating that when users believe that digital health systems are reliable and safe, they are more motivated to use them productively, thereby building their digital ability to use them further [74, 75]. This outcome is also in line with the study by Alam et al. [76], which found that trust was the strongest indicator of mHealth adoption by users in Bangladesh, and Zhao et al. [12] found a similar trend among users of telemedicine in China. Furthermore, Social influence predicted e‐health literacy and behavioral intention both significantly. This tendency is consistent with research indicating that recommendations from peers, relatives, and medical professionals strongly influence patient engagement with online services [30, 42]. The findings indicate that the social norms may operate in promoting the uptake of e‐health, besides encouraging users to develop literacy to utilize the tools with ease.

Performance expectancy is another critical factor that has direct and indirect impacts on e‐health adoption. Studies across diverse populations and settings (patients, older adults, and health professionals) show that when users perceive clear benefits, their willingness to adopt and use e‐health tools increases significantly [29, 33, 49, 77]. Performance expectancy also exerts indirect effects through mediators such as e‐health literacy, perceived ease of use, and attitude. Users who expect meaningful benefits are more likely to develop the skills and confidence needed to use digital health tools, which further enhances their intention to adopt [20, 78]. It's also observed in another study conducted in China utilizing the UTAUT model for examining the adoption of the Chinese m‐health platform [79].

Interestingly, perceived ease of use, a traditional core predictor in TAM, did not have a substantial direct impact on behavioral intention, while perceived usefulness was supported at a quasi‐significant level. These findings contradict the traditional findings, which identify perceived ease of use as a primary driver of technology adoption [80, 81, 82]. The nonsignificant results for perceived ease of use and perceived usefulness could be due to e‐health services like Shukhee, users' prior experience, and literacy, which could mitigate these direct effects. Moreover, healthcare technology adoption differs from technology acceptance, as users are more concerned with trust, privacy, and health outcomes than ease of use when deciding to use a health application [81, 83]. In other words, ease is not only required but also not a sufficient condition for adopting digital platforms in modern digital health circumstances, where most users already have some basic familiarity with them [78].

Lastly, facilitating conditions also had a strong prediction of behavioral intention but had no significant contribution to e‐health literacy. This observation implies that resource availability and support may not significantly enhance users' digital capabilities when using e‐health services, despite the fact that these factors contribute to users' adoption of the service.

The mediation analysis provides partial support for e‐health literacy as the mediator between users' psychological perceptions and their behavioral intention. Specifically, literacy significantly mediates the effects of perceived trust, social influence, and performance expectancy, indicating that literacy is an important mediator in understanding how these factors are translated into intention [67]. Furthermore [66], found that eHealth literacy is a direct predictor of attitudes toward artificial intelligence (AI) use among nurses, as well as having a relationship with trust in technology/perceived value via a chain‐mediation pathway, reaffirming the notion of literacy as a foundational element that prompts subsequent psychological processes leading to technology acceptance [84]. Collectively, these findings across a variety of healthcare or technology settings support that domain‐specific literacy is not only an antecedent of technology adoption but also a transformative capacity that influences how individuals process and respond to other factors that contribute to technology adoption [25]. These results continue previous research showing that literacy can enhance the confidence and autonomy of users of e‐health platforms [85]. These findings also align with previous literature indicating that literacy is a precondition for successful digital interaction, particularly the ability to navigate the complexity and variability of online health information [45].

## 5 Conclusion

This study examines the determinants of behavioral intention to use e‐health services and examines the mediating role of e‐health literacy within an integrated TAM‐UTAUT framework. The results indicate that perceived trust, social influence, performance expectancy, facilitating conditions, and effort expectancy have become important predictors. It is worth noting that e‐health literacy has a direct effect on intention and mediates the impact of critical predictors such as trust, social influence, and performance expectancy, but does not mediate the effects of perceived ease of use, perceived usefulness, effort expectancy, or facilitating conditions. These findings support the idea that the adoption of e‐health services is not only related to perceptions of ease or usefulness, but also to users' digital competencies and their confidence in operating in the digital health setting.

## 6 Implications

### 6.1 Theoretical Implications

The current study provides various additions to the e‐health adoption theory. First, it establishes e‐health literacy as a key construct of an integrated TAM and UTAUT models. Although previous studies have considered the applicability of literacy, this paper has empirically confirmed that literacy is a direct and mediating factor of behavioral intention determinant. Second, as they disclose that conventional predictors like Perceived Ease of Use and Perceived Usefulness have a stronger impact on literacy than intention does, the findings can be improved by adding to the existing theoretical framework: ease and usefulness can act as an upstream influence on users developing digital competencies, but it is not necessarily the upstream hypothesis that influencing intention.

Furthermore, the high levels of impact of trust and social influence demonstrate the relevance of social cognitive and relational influences in e‐health adoption models. These findings justify the development of UTAUT‐based models to capture the credibility, safety and social validation constructs of digital health technologies. Lastly, the research study shows the importance of incorporating literacy in mediation routes, which would guide future researchers to model the concept of adoption as a process by developing skills, building confidence, and reinforcing through social support.

### 6.2 Practical Implications

The results based on the study data, provide a straightforward recommendation to health practitioners, technology developers, and digital health institutions. Based on these findings, this study suggests that digital health platforms should focus on trust‐building mechanisms, including data security and clear privacy assurances. E‐health literacy education must be a part of digital health interventions. Tutorials, interactive learning modules, and user‐friendly design can bring a significant difference in terms of literacy and subsequently intention to adopt. While the study result supports its role, the suggestion to integrate literacy programs as a key factor for success is speculative and should be further explored in future research. Also, the social influence was identified as an adoption intention driver, suggesting that platforms may improve by adding social support, including peer support or family support, and collaboration with local health influencers. Besides, with the differences in the needs of users in different demographic groups, platforms must provide customizable features to suit different levels of technological skills and health concerns. Finally, policymakers must also be made aware of the necessity of digital health literacy and trust‐building efforts, including community training and legislative support on data protection to enable more people to access digital health services in an equitable manner. Although these recommendations are based on the findings of the study, they need to be further examined in order to establish their efficiency in improving adoption.

### 6.3 Policy Implications

Our study is observational and limited to Shukhee app users from Dhaka, the study result offer preliminary finding for the policymakers. With that caveat, trust and social influence are important drivers to the adoption of digital health services. Policymaking should focus on the initiatives that can increase user trust by establishing effective data privacy laws and transparency of digital health platforms. This may involve putting in place definite legal frameworks to the safe management of personal health information and having platforms of high standards of data protection. Since social influence is critical, as it is determined in the study, policymakers are advised to think about ways to advance community‐based health programs that will utilize trusted local figures (e.g., community leaders and health professionals) to promote the use of digital health. This may include empowering and training local influencers who can become promoters of digital health tools to make them perceived as trustworthy and accessible to more people. Moreover, governments can also incorporate digital health literacy in their public health education so that citizens are aware of the advantages and disadvantages of utilizing digital health services. These actions have a direct correlation with the results of this paper, and they have the potential to develop the trust and involvement in adopting digital health, especially within underserved populations.

### 6.4 Limitations and Future Research

Despite the valuable information in this research, there are some limitations that should be noted. One, a cross‐sectional design does not allow one to infer causality; longitudinal research would be used to identify the effects of literacy and intention to use e‐health services as users become more familiar with these platforms. Second, this study used a purposive sampling technique, which targets respondents who use or are familiar with digital health platforms, so the sample might not fully represent diversity across socioeconomic, cultural, and geographic subgroups; thus, generalizability is limited. The future study can consider the variation in the determinants of adoption usage between older adults, rural people, people with disabilities, and low‐income earners, who might have different barriers. Third, the research is on self‐reported behavioral intention to use digital healthcare services, which, although informative, does not reflect the actual use behavior. As a predictor of future behavior, it does not guarantee that participants will follow through with their intention. Future work should explore actual usage patterns through longitudinal studies or real‐time tracking of digital health app usage to provide a clearer picture of adoption rates.

## Author Contributions

**Shanjida Chowdhury:** writing – review and editing, supervision. **Farhana Ferdousi:** conceptualization, investigation, funding acquisition, writing – original draft, methodology, validation, visualization, writing – review and editing, formal analysis, software, project administration, data curation, resources. **Tahsin Farzana Jisun:** writing – review and editing, supervision.

## Funding

The authors have nothing to report.

## Consent

The authors have nothing to report.

## Conflicts of Interest

The authors declare no conflicts of interest.

## Transparency Statement

The lead author, Shanjida Chowdhury, affirms that this manuscript is an honest, accurate, and transparent account of the study being reported; that no important aspects of the study have been omitted; and that any discrepancies from the study as planned (and, if relevant, registered) have been explained.

## Data Availability

The data that support the findings of this study are available from the corresponding author upon reasonable request.

## 1

**TABLE A1:** Descriptive statistics, collinearity (VIF), and factor loadings (Outer loadings) of variables.

Construct	Items	Mean	Median	SD	Skewness	Kurtosis	VIF	Outer loadings
Perceived ease to use (PEOU)	PEOU01	3.19	3	1.26	−0.238	−0.885	1.509	0.774
	PEOU02	3.23	3	1.239	−0.306	−0.815	1.510	0.769
	PEOU03	3.19	3	1.301	−0.237	−0.99	1.615	0.802
Perceived usefulness (PU)	PU01	3.17	3	1.266	−0.212	−0.922	1.546	0.787
	PU02	3.18	3	1.283	−0.26	−0.932	1.587	0.798
	PU03	3.17	3	1.271	−0.233	−0.886	1.463	0.751
Performance expectancy (PE)	PE01	3.45	4	1.353	−0.53	−0.96	2.060	0.880
	PE02	3.38	4	1.364	−0.509	−0.979	2.059	0.873
	PE03	3.45	4	1.403	−0.507	−1.065	1.942	0.866
	PE04	3.24	3	1.21	−0.219	−0.756	1.444	0.744
Effort expectancy (EE)	EE01	3.24	3	1.243	−0.295	−0.829	1.488	0.768
	EE02	3.21	3	1.279	−0.225	−0.866	1.562	0.788
	EE03	3.19	3	1.224	−0.305	−0.761	1.432	0.742
	EE04	3.22	3	1.273	−0.172	−0.945	1.552	0.787
Social influence (SI)	SI01	3.43	4	1.399	−0.474	−1.102	2.200	0.857
	SI02	3.41	4	1.347	−0.465	−1.027	2.225	0.859
	SI03	3.45	4	1.396	−0.559	−0.976	2.201	0.853
Facilitating conditions (FC)	FC01	3.21	3	1.29	−0.268	−0.921	1.598	0.796
	FC02	3.17	3	1.243	−0.133	−0.857	1.557	0.790
	FC03	3.16	3	1.262	−0.161	−0.866	1.531	0.770
Perceived trust (PT)	PT01	3.17	3	1.314	−0.364	−0.962	1.550	0.719
	PT02	3.17	3	1.327	−0.33	−0.996	1.640	0.754
	PT03	3.14	3	1.284	−0.285	−0.88	1.476	0.699
	PT04	3.14	3	1.341	−0.286	−1.03	1.796	0.783
	PT05	3.15	3	1.318	−0.279	−0.954	1.587	0.733
E‐health literacy (EL)	EL01	3.41	4	1.276	−0.474	−0.86	1.916	0.758
	EL02	3.53	4	1.263	−0.654	−0.576	1.829	0.741
	EL03	3.45	4	1.309	−0.615	−0.731	1.729	0.714
	EL04	3.52	4	1.246	−0.569	−0.696	1.731	0.715
	EL05	3.47	4	1.284	−0.535	−0.786	1.784	0.733
	EL06	3.35	4	1.318	−0.405	−1.025	2.038	0.778
	EL07	3.41	4	1.3	−0.526	−0.828	1.939	0.757
	EL08	3.42	4	1.304	−0.542	−0.84	1.864	0.747
	EL09	3.39	4	1.309	−0.525	−0.871	1.889	0.751
Behavioral intention to use (BI)	BI01	3.43	4	1.395	−0.579	−0.987	1.971	0.840
	BI02	3.54	4	1.304	−0.64	−0.755	1.825	0.820
	BI03	3.44	4	1.325	−0.581	−0.763	1.790	0.815

**TABLE A2:** Cross‐loadings of study variables.

	BI	EL	EE	FC	PEOU	PT	PU	PE	SI
BI01	**0.840**	0.689	0.648	0.672	0.658	0.639	0.670	0.704	0.700
BI02	**0.820**	0.705	0.632	0.647	0.639	0.640	0.631	0.666	0.689
BI03	**0.832**	0.679	0.651	0.659	0.635	0.636	0.649	0.692	0.721
EE01	0.598	0.592	**0.768**	0.580	0.619	0.471	0.568	0.581	0.591
EE02	0.589	0.627	**0.788**	0.618	0.548	0.483	0.598	0.625	0.628
EE03	0.561	0.562	**0.742**	0.630	0.540	0.444	0.557	0.575	0.575
EE04	0.627	0.592	**0.787**	0.596	0.558	0.489	0.603	0.608	0.658
EL01	0.601	**0.758**	0.556	0.550	0.584	0.562	0.577	0.639	0.620
EL02	0.587	**0.741**	0.563	0.595	0.526	0.503	0.586	0.587	0.632
EL03	0.590	**0.714**	0.509	0.562	0.543	0.554	0.541	0.579	0.626
EL04	0.615	**0.715**	0.582	0.551	0.570	0.510	0.576	0.587	0.603
EL05	0.620	**0.733**	0.556	0.618	0.592	0.531	0.567	0.618	0.632
EL06	0.669	**0.778**	0.651	0.587	0.605	0.605	0.644	0.662	0.662
EL07	0.626	**0.757**	0.582	0.610	0.586	0.560	0.603	0.650	0.644
EL08	0.627	**0.747**	0.593	0.596	0.613	0.563	0.616	0.620	0.624
EL09	0.612	**0.751**	0.552	0.602	0.582	0.586	0.549	0.634	0.646
FC01	0.638	0.627	0.630	**0.796**	0.592	0.521	0.636	0.673	0.643
FC02	0.663	0.642	0.646	**0.790**	0.612	0.536	0.594	0.647	0.655
FC03	0.588	0.593	0.609	**0.770**	0.609	0.460	0.579	0.598	0.626
PE01	0.756	0.741	0.707	0.743	0.684	0.636	0.696	**0.880**	0.749
PE02	0.698	0.713	0.656	0.711	0.679	0.650	0.688	**0.873**	0.742
PE03	0.727	0.728	0.664	0.693	0.674	0.635	0.690	**0.866**	0.765
PE04	0.678	0.703	0.656	0.711	0.679	0.650	0.688	**0.843**	0.731
PEOU01	0.608	0.611	0.555	0.579	**0.774**	0.557	0.569	0.581	0.633
PEOU02	0.600	0.595	0.593	0.606	**0.769**	0.463	0.567	0.614	0.627
PEOU03	0.631	0.632	0.583	0.602	**0.802**	0.531	0.629	0.630	0.645
PT01	0.547	0.538	0.452	0.484	0.507	**0.719**	0.492	0.545	0.501
PT02	0.600	0.559	0.494	0.533	0.515	**0.754**	0.521	0.556	0.556
PT03	0.535	0.534	0.406	0.462	0.454	**0.699**	0.465	0.509	0.497
PT04	0.611	0.571	0.480	0.499	0.530	**0.783**	0.533	0.570	0.538
PT05	0.576	0.552	0.431	0.484	0.473	**0.733**	0.487	0.540	0.548
PU01	0.634	0.616	0.565	0.582	0.585	0.551	**0.787**	0.627	0.633
PU02	0.631	0.637	0.586	0.551	0.598	0.535	**0.798**	0.616	0.656
PU03	0.566	0.576	0.581	0.603	0.573	0.466	**0.751**	0.581	0.648
SI01	0.723	0.739	0.691	0.703	0.682	0.611	0.703	0.728	**0.857**
SI02	0.724	0.743	0.689	0.707	0.730	0.616	0.712	0.760	**0.859**
SI03	0.713	0.692	0.696	0.679	0.698	0.585	0.700	0.706	**0.853**

*Note:* Bold values represent the indicators outer loading on it's respective construct.
